# Interacting with chatbots later in life: A technology acceptance perspective in COVID-19 pandemic situation

**DOI:** 10.3389/fpsyg.2022.1111003

**Published:** 2023-01-16

**Authors:** Ioana Iancu, Bogdan Iancu

**Affiliations:** ^1^Department of Communication, Public Relations, and Advertising, Babeș-Bolyai University, Cluj-Napoca, Romania; ^2^Computer Science Department, Technical University of Cluj-Napoca, Cluj-Napoca, Romania

**Keywords:** chatbots, technology acceptance model, middle-aged and aging adults, perceived ease of use, perceived usefulness, behavioral intention

## Abstract

**Introduction:**

Within the technological development path, chatbots are considered an important tool for economic and social entities to become more efficient and to develop customer-centric experiences that mimic human behavior. Although artificial intelligence is increasingly used, there is a lack of empirical studies that aim to understand consumers’ experience with chatbots. Moreover, in a context characterized by constant population aging and an increased life-expectancy, the way aging adults perceive technology becomes of great interest. However, based on the digital divide (unequal access to technology, knowledge, and resources), and since young adults (aged between 18 and 34 years old) are considered to have greater affinity for technology, most of the research is dedicated to their perception. The present paper investigates the way chatbots are perceived by middle-aged and aging adults in Romania.

**Methods:**

An online opinion survey has been conducted. The age-range of the subjects is 40–78 years old, a convenience sampling technique being used (*N* = 235). The timeframe of the study is May–June 2021. Thus, the COVID-19 pandemic is the core context of the research. A covariance-based structural equation modelling (CB-SEM) has been used to test the theoretical assumptions as it is a procedure used for complex conceptual models and theory testing.

**Results:**

The results show that while perceived ease of use is explained by the effort, the competence, and the perceive external control in interacting with chatbots, perceived usefulness is supported by the perceived ease of use and subjective norms. Furthermore, individuals are likely to further use chatbots (behavioral intention) if they consider this interaction useful and if the others’ opinion is in favor of using it. Gender and age seem to have no effect on behavioral intention. As studies on chatbots and aging adults are few and are mainly investigating reactions in the healthcare domain, this research is one of the first attempts to better understand the way chatbots in a not domain-specific context are perceived later in life. Likewise, judging from a business perspective, the results can help economic and social organizations to improve and adapt AI-based interaction for the aging customers.

## Introduction

The COVID-19 pandemic situation has transformed technology into a focal point. From work-from-home situations to remote education and remote communication, the pandemic forced individuals, regardless of their cognitive, affective, and behavioral profile, to adopt all types of technologies that have been rapidly developed and adapted. Although the technological solutions existed before the crisis, the rhythm of implementing them increased exponentially. Likewise, since the beginning of the pandemic, there is an increasing pressure on the healthcare system, especially dedicated to aging population, and, thus, digital solutions are intensively searched for (Valtolina and Marchionna, 2021). Artificial Intelligence (AI) is considered as one of the most important priorities when it comes to investment (Sheth et al., 2019). In this respect, chatbots’ market is expected to growth to USD 24.98 billion with a 24.2% Compound Annual Growth Rate (CAGR) by 2030 (Market Research Report, 2022).

Within the technological development path, chatbots are an important tool for companies to become more efficient (Canhoto and Clear, 2020), to create a more personalized digital experience, and to develop “customer-centric” experiences that mimic human behavior (Toader et al., 2020). Moreover, chatbots are increasingly used for healthcare purposes (Tamamizu et al., 2017; Mesbah and Pumplun, 2020; Zhang and Zheng, 2021), as they support the independence of aging adults, reduce the burden on caregivers, have the capacity to make people talk more honestly (Miura et al., 2022), and are effective in increasing the conversation time (Ryu et al., 2020).

Being built based on AI’s features, chatbots are considered intelligent entities that understand verbal, written, or multimodal communication, that are programmed to semantically respond, using natural conversational language, and that can learn from past experiences to improve themselves (Sheth et al., 2019; Toader et al., 2020). Although chatbots are already usually found in the online retail domain, their presence is increasingly acknowledged in the healthcare field (Valtolina and Marchionna, 2021). Thus, the most common chatbot applications are in domains as healthcare, e-commerce / customer services, education, financial and banking services (Bächle et al., 2018; Toader et al., 2020; Alt et al., 2021), or tourism (Melián-González et al., 2021).

The changes brought by the technological development have led to fundamental changes in the interaction between economic or social entities and consumers (Toader et al., 2020). Thus, studying chatbots’ impact on individuals’ perception becomes of great importance. Although AI technologies are increasingly used in interactions with customers, from pre-purchase to service support, there is a lack of empirical studies that aim to understand consumers’ experience with AI in general, and with chatbots in particular (Ameen et al., 2020; Nichifor et al., 2021). Likewise, most of the studies are in the computer science domain and are technically testing chatbots’ prototypes.

Moreover, in social sciences, chatbot studies on the Romanian context are limited. The existing ones are mainly focused on the relationship between chatbots’ error and gender, social presence, perceived competence, anthropomorphic design and trust in the digital marketing domain (Toader et al., 2020), on the acceptance of digital banking services (Alt et al., 2021; Schipor and Duhnea, 2021), on electronic commerce (Nichifor et al., 2021), or on marketing communication (Popescu, 2020). Yet, a not domain-specific approach that considers regular chatbots used in all types of daily online interactions might add value to the already existing research.

Simultaneously with the technological development, one of the most significant social transformations of the twenty-first century is the population aging. Globally, persons aged 65 or above have outnumbered children under 5 years old and it is estimated that by 2050, there will be around 2.1 billion aging adults worldwide (United Nations (UN), 2022). In the case of Romania, the average age is already 42 years old, and the most numerous age-range is 50–54 years old (Dan, 2022). Furthermore, it is estimated that by 2050, 60% of the population will be over 65 years old (Coman, 2021) and, thus, loneliness and isolation are predicted to deepen (Da Paixão Pinto et al., 2021). This reality is believed to increase both the economic and social pressure, as there is a rise in public health expenditure (Chen and Schulz, 2016; Fang and Chang, 2016; Segercrantz and Forss, 2019), a permanent need for improved healthcare assistance and assistive living, and a scarce of providers (Hofmann, 2013; Bassi et al., 2021). Low income and high workload generate a shortage of caregivers (Yang et al., 2015).

Thus, technology is believed to solve the gap between the needs of aging population and the potential of the society to overcome them, to prevent isolation, to communicate, to interact, and to monitor (Niehaves and Plattfaut, 2014; Petrie et al., 2014; Huh and Seo, 2015). In this context, an improved quality of life means smart medical care, virtual companion, mental health monitoring, open-ended conversations, emotional and knowledge-based responses, reminders, notifications, or financial duties (Bassi et al., 2021).

Baby boomers (people between 57 and 75 years old) and generation X (people between 42 and 56 years old) are considered “*digital illiterates*” (Vasilateanu and Turcus, 2019). As this dichotomy is too sharp, Lenhart and Horrigan (2003) consider that a digital spectrum approach is more correct. Relying on the fact that each generation has its technology laggards, on the fact that aging adults are not a homogenous cohort in terms of technology use, age should be correlated with other variables, as education or frequency of use of a certain application or device (Loos, 2012). Based on the digital divide (unequal access to technology, knowledge, and resources; DiMaggio et al., 2001), since young people are considered to have a greater affinity for technology, most of the research is dedicated to the perception of Generation Z and Millennials (people between 18 and 34 years old; Nichifor et al., 2021; Schipor and Duhnea, 2021). Thus, unfairly, the views of middle-aged individuals and aging adults on technology are receiving less attention (Nikou, 2015). Digital divide should rather be understood as relative rather than absolute inequalities that can be reduced (Van Dijk and Hacker, 2003). Furthermore, as the literature highlights a varied range of technologies that can improve aging adults’ lives, their perception on technology is rarely assessed (Pal et al., 2018). Since the technology developers are usually young people, a gap between what is invented and what is needed appears (Lee and Coughlin, 2014). Although chatbots are designed to be able to identify health problems based on the exposed symptoms, such applications are mainly restricted to young generations (Mesbah and Pumplun, 2020). Thus, the aging adults’ user experience should be better understood for more suitable innovations.

To the best of our knowledge, there is a lack of studies that focus on the general use of chatbots by aging population, without particularly emphasizing the healthcare or assistive domains. One of the newest paper in this respect is a systematic literature review written by Da Paixão Pinto et al. (2021) in which authors are interested in the engagement strategies of the chatbots, in their computational environments, in the input data format accepted, in the different types of personalization offered, and in the evaluation techniques for conversational agents. Being based on a systematic analysis of 53 papers, the main results of the study emphasize that personalization, context adaptation, a speech type input, and an intuitive system are at the core of an increased engagement and interaction with chatbots (Da Paixão Pinto et al., 2021). However, based on the existing findings,, further empirical investigation is needed.

Based on the existing literature, we find that chatbots are mainly studied from a computer science perspective, on a young audience, or with a strong focus on healthcare domain. Thus, the present paper aims to empirically deepen the social-science knowledge on chatbots both by understanding the middle-aged and aging adults’ perceptions on chatbots in Romania and by analyzing possible determinants of behavioral intention on using chatbots in a not domain-specific perspective. An online opinion survey has been conducted on a convenience sample (*N* = 235), aged between 40 and 78 years old (*M* = 51.13, *SD* = 5.954). Although aging adults, or elderly, are persons aged 60 years old and above (United Nations (UN), 2017), due to the limitations given by the convenience sampling procedure, this study extends the analyzed age range and aims to comparatively investigate the differences, if any, between middle-aged adults and aging ones. The survey has been applied in the COVID-19 pandemic situation, between May and June 2021. By relying on Technology Acceptance Model (Davis, 1989) and on its extended version (Venkatesh and Bala, 2008), the respondents are mainly inquired on the general perceived ease of use, perceived usefulness, enjoyment, competence, effort, pressure, satisfaction, perception of external control, subjective norms, and behavioral intention, all related to the use of chatbots.

Based on Digital Economy and Society Index (2022) report, Romania is ranked as the last European Union (EU) country on digital skills and with a poor performance regarding the integration of digital technologies and digital public services. Furthermore, among developing countries, Romania is considered to be a case in which aging adults are the latest technology adopters (Ivan and Cutler, 2021). This situation has been deepened by the COVID-19 pandemic (Motorga, 2022). Considering this poor digital literacy context, Romania becomes a significant case-study on which technological development and adoption is urgently needed.

The implications of the paper are at least threefold. First, the present study aims to enrich the existing literature with a technology acceptance overview on the way chatbots are perceived, regardless of the domain. Interestingly, in comparison with the results of other studies testing technology acceptance models, the present data show that some variables (e.g., enjoyment, satisfaction, etc) do not have the hypothesized effect in explaining behavioral intention in respect to chatbot interaction. Explanations might be related to the target group of the study (which is different than most of the samples used in similar context), or to their understanding and experience with chatbots. Thus, the results open new research perspectives for verifying the present model’s outcomes. Second, the research fulfills the existing gap on the target group. Since most of the studied samples are composed of young people, the present approach focuses on middle-aged and aging adults. Finally, judging from a business perspective, the results can help economic and social organizations to improve and adapt AI-based interaction for the aging customers.

### Chatbots

Chatbots are also known as chatterbots (Miliani et al., 2021), smart bots or interactive agents (Adamopoulou and Moussiades, 2020), virtual assistants or conversational agents (Sheth et al., 2019). They are chatty software machines that, based on artificial intelligence features and natural language processing, interact with users using written text or spoken language (Bächle et al., 2018) and relying on image, video, and audio processing (Bala et al., 2017). Put it differently, a chatbot is a computer program designed to interact through a natural dialog with users and to create the sensation of communicating with a human being (Hussain et al., 2019). Conversational agents are either on-screen or as voice assistants (Gunathilaka et al., 2020).

When Alan Turing proposed the Turing Test [in which users are tested if they are capable to differentiate between an interaction with a human being or a machine (Chen et al., 2020)], starting from the question “*if machines can think?*,” the idea of chatbots started to spread (Adamopoulou and Moussiades, 2020). Chatbot ELIZA, a simulation of a person-centric psychotherapist, is the first chatbot attempt. It was developed in 1966 by Josepth Weizenbaum and it used word and pattern matching techniques to conduct simple conversation (Bächle et al., 2018; Nichifor et al., 2021). ELIZA program used to search for keywords within the user’s input and transform the sentence into a script (Hussain et al., 2019). In the 80’s, chatbots have been mainly developed for the gaming industry and they have been used for testing if individuals can recognize them as being machines or humans (Bächle et al., 2018). Although early chatbots lacked the ability to maintain a conversation going (Hussain et al., 2019), as the conversational agents use more and more natural language processing, they pass the Turing Test (Justo et al., 2021). In 1995, ALICE chatbot, a highly awarded software, has been developed and has been considered the “most human computer” until that moment (Adamopoulou and Moussiades, 2020). After the development of chatbots available through messenger application, like SmarterChild, in 2001, or Wechat in 2009 (Mokmin and Ibrahim, 2021), the creation of virtual personal assistance has begun (e.g., Siri form Apple, Cortana from Microsoft, Alexa from Amazon, Google Assistant, or IBM Watson; Liu et al., 2018; Adamopoulou and Moussiades, 2020; Gunathilaka et al., 2020).

Chatbots can be either task-oriented or non-task-oriented (Hussain et al., 2019). Task-oriented chatbots are created for very specific tasks and domain-based conversations. Examples in this respect are booking accommodation, booking a flight, placing an order in online shopping, accessing specific information, etc (Hussain et al., 2019). The drawback of a task-oriented system is that it cannot exceed the programmed topic scope (Su et al., 2017). Non-task-oriented chatbots is rather keen on conversating for entertainment purpose in all kinds of domains and in an unstructured manner (Hussain et al., 2019; Justo et al., 2021).

As chatbots are imitating human-to-human interaction, they are often perceived as anthropomorphic (Seeger et al., 2018). Moreover, they are one of the most used examples of intelligent human-computer interaction (Adamopoulou and Moussiades, 2020). The literature talks about the capability of chatbots to expand beyond repetitive tasks (mechanically intelligent AI) and conduct thinking tasks (analytical intelligent AI), creative tasks (intuitive intelligent AI), and feeling tasks (empathetic intelligent AI). While mechanic chatbots provide predefined responses, the analytical chatbots analyze the given problems, intuitive chatbots contextually understand complains, and emphatic chatbots recognize and understand users’ emotions (Youn and Jin, 2021).

While the technological development’s aim is that of creating realistic human-like chatbots, in comparison with an employee, a chatbot is constantly updating, has unlimited memory, acts instantly, and it is available all the time (Lo Presti et al., 2021). The most important features of chatbots are their capability of being self-contained, always active, and able to track users’ interest, preferences, and socio-demographics (Tascini, 2019). Chatbots can be used for customer services, allowing companies to target consumers in a very personalized way (Alt et al., 2021) and expanding satisfaction and engagement (Maniou and Veglis, 2020). They are considered a technological trend for the companies as they can speed up and facilitate customer service process through providing online information or placing orders in real time (Ashfaq et al., 2020; Nichifor et al., 2021). Chatbots allow users to interact online with different organizations, anytime and from any place, and offer quick and meaningful responses (Alt et al., 2021). A useful chatbot is responsible to provide assistance without interfering, and developing a sense of trust (Zamora, 2017).

The functionality of chatbots is either for entertainment or utilitarian (Zamora, 2017). Conversational systems are increasingly used and are useful both at home and for leisure (e.g., Alexa, Siri), or in our professional life (e.g., Siri, Cortana) for managing the schedule or for educational purposes (Justo et al., 2021; Valtolina and Hu, 2021). Chatbots are mainly used for obtaining information, for interacting needs and out of curiosity (Gunathilaka et al., 2020).

Studies have revealed that chatbots perform better is they are specifically created for a certain domain or group (De Arriba-Pérez et al., 2021). Chatbots can be used in domains such as e-commerce, business, marketing, communication, education, news, health, food, design, finance, entertainment, travel, or utilities, but not limited to them (Liu et al., 2018; Adamopoulou and Moussiades, 2020). Technology is undoubtedly perceived as being the solution for improving healthcare. Scholars that develop chatbots talk about the need of designing empathetic virtual companions to alleviate isolation and loneliness (Da Paixão Pinto et al., 2021) and to fulfill the emotional needs of the aging adults and to increase likeability and trustworthiness towards machines (Yang et al., 2015). As the main motivation to use chatbot is productivity, together with entertainment and socialization, chatbots should be equally built as a tool, a toy, and a friend (Brandtzaeg and Følstad, 2017).

The literature also emphasizes on the downsides of chatbots use, especially on the reluctance on interacting with an impersonal machine instead of a human being (Nichifor et al., 2021). Although chatbots are increasingly resembling humans, this can be perceived as a disadvantage since privacy and security issues are associated with human hackers (Michiels, 2017). At the same time, while programmed with natural language processing, the interaction with chatbots is not intuitive enough and might imply errors. Thus, the lack of human feelings and emotions echoes on the lack of engagement and personality (Knol, 2019). For instance, in a shopping context, most of the users feel uncomfortable receiving personalized feedback from chatbots and consider them as being immature technology (Rese et al., 2020; Smutny and Schreiberova, 2020). Hildebrand and Bergner (2019) highlight the possibility that a chatbot service interaction provided to an already disappointed consumer might have a deep negative effect on both the service value and the brand or the organization. Furthermore, while users might have limited knowledge on chatbots and might consider them as inferior and unworthy entities to communicated with, the feeling of discomfort can lead to the refusal of interaction (Ivanov and Webster, 2017). When it comes to elderly, chatbots are associated with privacy issues, with loss of autonomy, with technical fears, or with lack of usefulness that can increase their resistance and avoidance (Da Paixão Pinto et al., 2021). Thus, although useful for organizations, there are many variables that can deter a good communication flow between chatbots and users. While, on one hand, there are the features of the chatbots (e.g., the way they are designed, their cognitive capabilities, etc), on the other hand there are the variables affecting the perception on them (e.g., knowledge on and experience with technology, the need for a human-natural conversation, or usefulness).

### Technology acceptance, chatbots and aging

Renaud and Van Biljon (2008) talk about three main reasons for technology adoption. First, there is the support given for certain activities, as information, communication, administrative, entertainment, or health monitoring. Second, there is the convenience reason, referring to reducing physical and mental endeavor. Finally, there are the technology features, namely the design of the device, specific actions, and options. The attitude toward technology, measured on the strength of how much an individual likes or dislikes it, is also believed to be a key factor in accepting and adopting a particular technology (Edison and Geissler, 2003).

The most referred to theory on technology acceptance is Technology Acceptance Model (TAM; Davis, 1989). Aiming to predict behavior, this theory is inspired from the Theory of Reasoned Action (TRA; Fishbein and Ajzen, 1975) and the Theory of Planned Behavior (TPB; Ajzen, 1985). TAM emphasizes that perceived ease of use and the perceived usefulness, together with other external variables, can predict the attitude towards using a certain technology, the intentional behavior, and, finally, the actual behavior (Davis, 1989; Renaud and Van Biljon, 2008; Minge et al., 2014; Alt et al., 2021). While perceived ease of use is defined as the degree to which using a particular device or system is free from effort, perceived usefulness is the degree to which using a certain device enhances one’s performance (Davis, 1989; Wang and Sun, 2016). Although perceived usefulness is considered as being a stronger predictor for the intentional behavior, perceived ease of use is a key variable for the initial acceptance (Lin et al., 2007; Renaud and Ramsay, 2007). Behavioral intention, as a strong predictor of the actual behavior, is defined as the strength of one’s aim to execute a specific behavior (Fishbein and Ajzen, 1975). Furthermore, as a certain device or application is easy to use, it is predicted that this perception is likely to influence the perceived usefulness (Venkatesh and Bala, 2008).

One of the most complex models that aims to explain technology acceptance is TAM3 (Venkatesh and Bala, 2008). Being developed in a managerial context and on a longitudinal perspective, the new model builds on the anchoring and adjustment framing of human decision and adds new variables and connections to the previous models. In the context of decision making, anchoring refers to relying on the available initial information, information that can further influence the decision but that will decline over time when adjustment knowledge will be accessible (Cohen and Reed, 2006; Qiu et al., 2016). Thus, the anchor variables, as device self-efficacy [perceived abilities to perform a specific task using a certain technology (Compeau and Higgins, 1995)], perception of external control [perceived control and resources on using a certain technology (Venkatesh et al., 2003)], and device anxiety (fear on using a certain technology) are considered as influencing the perceived ease of use (Venkatesh and Bala, 2008).

The same relationship is developed when it comes to adjustment variables as perceived enjoyment on the use of a certain technology and objective usability (the effort required to interact with a certain technology; Venkatesh, 2000). The literature stresses on some universal incentives that can motivate individuals and that can predict intention to use a certain technology. While on one hand, there are the utilitarian rewards, as achievements, or ease of use, on the other hand, there are the hedonic rewards, as enjoyment and entertainment (Kim et al., 2018; Van Roy et al., 2018). Relying on Uses and gratification theory (Blumler and Katz, 1974), users tend to accept a device or an application if they feel rewarded (in terms of knowledge, relaxation, escapism, or social interaction) by using it (Kim et al., 2018; Wang et al., 2018). Likewise, an enjoyable experience with the technology is becoming increasingly infused into acceptance decision (Deng, 2017; Tsoy, 2017). Strongly linked with enjoyment, satisfaction is also considered as an import variable that can positively influence the perceived ease of use of a certain device or application (Zamora, 2017). At the same time, subjective norms, defined as the degree to which users consider that people in their trust circle should use a certain technology (Venkatesh and Davis, 2000), are believed to influence the perceived usefulness and behavioral intention.

Especially affected by the pandemic, but even beyond it, aging adults’ lives are usually characterized by loneliness and isolation. To avoid mental issues, like depression and anxiety, they need to be engaged into the daily routine, to be stimulated and entertained (Valtolina and Hu, 2021). Social interaction is considered a basic need for aging population in which solitude is one of the biggest issues (De Arriba-Pérez et al., 2021). Like in a vicious circle, social isolation, rejection, or loneliness seems to have a paramount negative effect on the general and mental health of an individual. Thus, assistive technology, by its social interaction capabilities and engagement, can help in preventing illnesses (Gunathilaka et al., 2020) and offers a more comfortable and cost-effective medical care (Mesbah and Pumplun, 2020). However, although technology can solve this problem, aging adults are resistant to change (Vichitvanichphong et al., 2017) and the digital divide is a barrier (De Arriba-Pérez et al., 2021). Considering that many devices and applications are created without considering users’ perception and are designed by young people, many aging adults are reluctant to products they do not understand (Lee and Coughlin, 2014; Pelizäus-Hoffmeister, 2016). In this respect, the experience with a certain technology is a key moderator variable for perceived ease of use and perceived usefulness relationship, for the technology anxiety and perceived ease of use, and for the perceived ease of use and behavioral intention (Venkatesh and Bala, 2008). In the present context, the previous use of chatbots (Luo and Remus, 2014), the previous knowledge on them (Cui and Wu, 2019), the initial attitude on them (Hall et al., 2017), and how it is liked might be important variables.

Some studies, by offering a generalized perspective, claim that aging adults perceive themselves as being too old to learn how to use technology (Feist et al., 2010). They are having less experience with devices, have fewer specific skills (Damant and Knapp, 2015), and the feelings of helplessness are being reinforced by failed previous experiences (Minge et al., 2014). In the case of aging population, some of the main largely accepted motivations to learn to use technology are social integration, usefulness, and security (Chou and Liu, 2016; Wang et al., 2018). Thus, being helpful and fulfilling needs and expectations become paramount variables (Gatti et al., 2017).

When it comes to chatbots, most of the studies are relying on young samples. Moreover, the literature review reveals that almost all papers on the use of chatbots for aging population are related to the healthcare or assistive domain. Almost all of them are written from a technical point of view by computer science specialists. Although AI developments in assistive technology are advanced, there is still work to be done for achieving a proper chatbot for end aging users (De Arriba-Pérez et al., 2021). Thus, the existing studies refer to presenting, designing, and testing prototypes of conversational agents with meaningful, empathetic emotional, and friendship capabilities (Yang et al., 2015; Bassi et al., 2021), with personalized entertainment content access (De Arriba-Pérez et al., 2021), with workplace environment facilities (Bächle et al., 2018), with capabilities to promote healthy habits through a coaching model (Justo et al., 2021), with public administration abilities (Miliani et al., 2021), with virtual caregiving attributions (Su et al., 2017; Ryu et al., 2020; Valtolina and Hu, 2021; Miura et al., 2022).

In a systematic literature review on general use of chatbots by aging adults, Da Paixão Pinto et al. (2021) have found that, considering the innovations in natural language processing, speech is the most used and preferred input format for aging adults to interact with chatbots. They also emphasize that assistive conversational agents still face acceptance problems, low involvement, and low user satisfaction mainly due to the loss of autonomy, privacy, and technical errors (Da Paixão Pinto et al., 2021).

Based on a set of interviews on assistive living with long term patients in Sri Lanka, Gunathilaka et al. (2020) have found that, due to poor sight, voice-based conversational agents can be helpful for long-term patient care if they are specialized for specific requirements, in accordance with individuals’ needs. However, although virtual agents can help aging adults to be more independent and better enjoy the lonely moments, the devices cannot substitute a human caregiver especially from an emotional point of view (Gunathilaka et al., 2020).

A comprehensive study on aging adults’ acceptance of health chatbots is using the extended Unified Theory of Acceptance and Use of Technology (UTAUT2) model (Venkatesh et al., 2012) to qualitatively test the factors that contribute to the adoption intention (Mesbah and Pumplun, 2020). Beside the interviews, the respondents have tested a health chatbot for a better understanding of the technology. The results show that behavioral intention to use a health chatbot depends not only on the performance expectancy, effort, social influence, facilitating conditions, as the initial model states, but also on variables as patience, resistance to change, need for emotional support, technology self-efficacy and anxiety, privacy risk expectancy, or trust in the technology and in the recommendation of a chatbot (Amato et al., 2017; Mesbah and Pumplun, 2020). As trust is especially considered an important factor in technology acceptance, it is predicted to positively impact the intention to use a certain technology, the perceived ease of use and the perceived usefulness when it comes to e-commerce and e-services (Gefen and Straub, 2003; Pavlou, 2003; Lankton et al., 2015; Cardona et al., 2021).

When it comes to gender, technology acceptance is debatable. While some studies consider that there are no significant relationships between gender and computer attitude (Nash and Moroz, 1997), men tend to score higher than women in affinity for technology (Edison and Geissler, 2003). Gender is usually considered as a moderator variable within the technology acceptance models (Bagana et al., 2021). Although the difference between men and women is narrow, men are believed to experience a lower level of technology anxiety (Damant and Knapp, 2015) and thus a higher behavioral intention.

Based on the above-described literature, the hypotheses of the paper are listed below:

*H1*: Perceived ease of use of chatbots is positively impacted by enjoyment (H1a), satisfaction (H1b), effort (H1c), competence (H1d), pressure (H1e), and perception of external control (H1f).

*H2*: Perceived usefulness of a chatbot is positively impacted by the perceived ease of use (H2a) and the subjective norms (H2b).

*H3*: Behavioral intention to use a chatbot in the future is positively impacted by the perceived usefulness (H3a), perceived ease of use (H3b), subjective norms (H3c), and previous experience with a chatbot [knowledge on chatbots (H3d), hearing about chatbots (H3e), use of chatbots (H3f), and like chatbots (H3g)].

*H4*: Men in comparison with women (H4a), and middle-aged in comparison with aging adults (H4b) are more likely to report a higher level of behavioral intention to use chatbots in the future.

**Figure 1:**
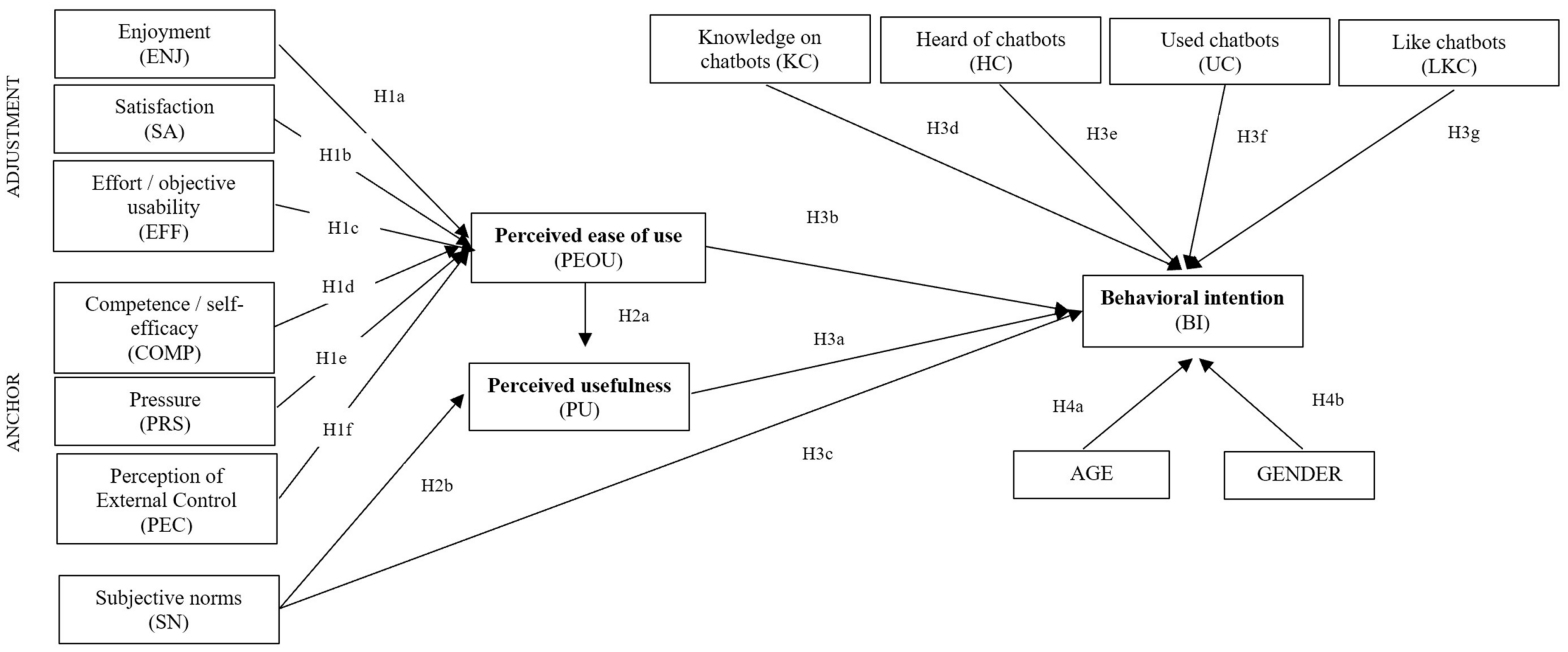
The structural model.

The schematic version of the structural model is presented in the Figure 1.

## Methodology

### Procedure

**Figure 2:**
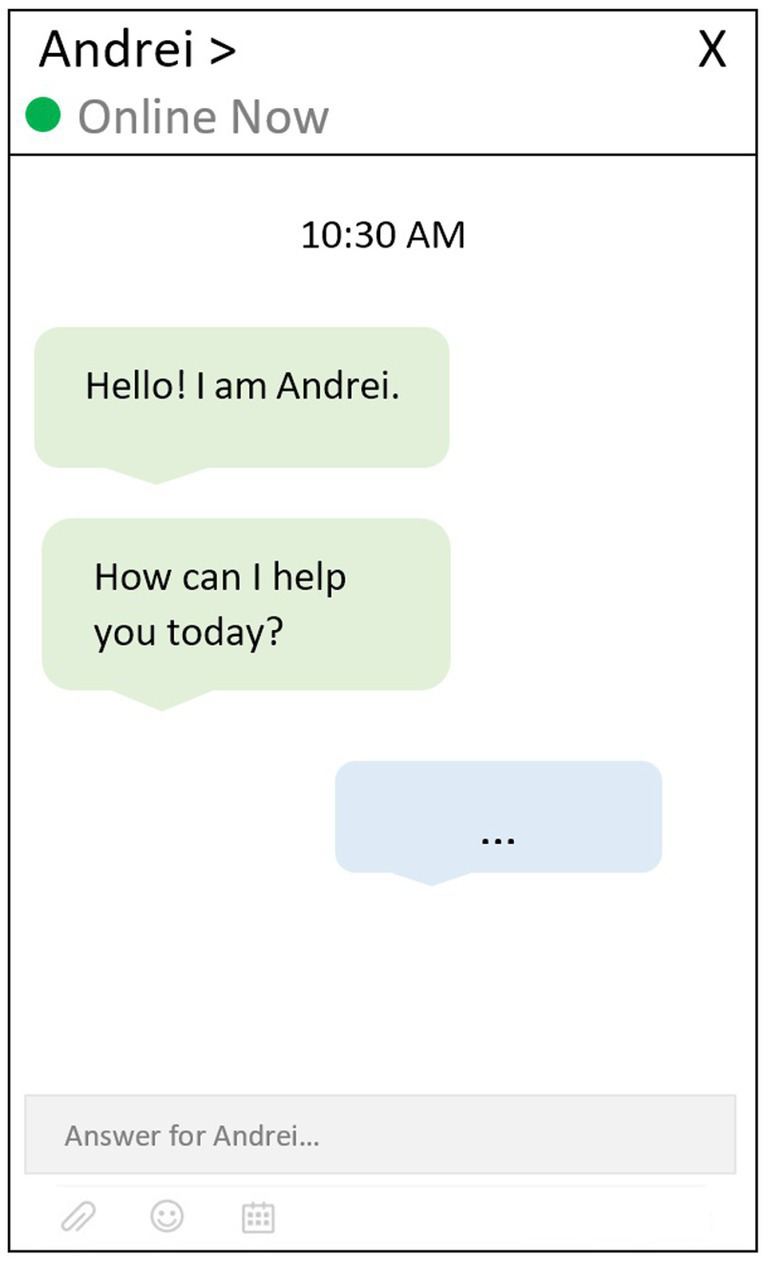
An example of chatbot interaction given within the survey.

To assess the above-mentioned relationships, an online opinion survey has been conducted. The questionnaire has been designed in Google Forms and it has been self-administrated during COVID-19 pandemic, namely between May and June 2021. Considering that the sample is formed of people over 40 years old and to avoid discrepancies due to communication difficulties (Cardona et al., 2021), the questionnaire has been developed in Romanian language. For a clear understanding of the research scope, the questionnaire has an introduction part in which the aim of the survey is presented together with information about the anonymity and confidentiality of the data. Moreover, for a more accurate understanding of a chatbot interaction, a small example has been given at the beginning of the survey (Figure 2). Andrei is the chosen name for the chatbot, as it is one of the most familiar names in Romania.

The questionnaire is composed of three main sections. The first section evaluates the previous experience with chatbots (if the users have heard of chatbots, of they use them, if they have knowledge on them, and if they like interacting with them). At the same time, the situations in which chatbots have been used and their perceived benefits are inquired. The second section highlights the main variables of technology acceptance models: perceived ease of use, perceived usefulness, enjoyment, satisfaction, effort, competence, pressure, perception of external control, subjective norms, and behavioral intention. The last section is dedicated to the socio-demographical variables.

A covariance-based structural equation modelling (CB-SEM) has been used to test the theoretical assumptions as it is a procedure used for complex conceptual models (Nitzl, 2016; Hair J. et al., 2019) and theory testing (Hair J. et al., 2019; Hair J. F. et al., 2019). The data has been analyzed using IBM SPSS and Amos 26 version.

### Sample

The analyzed sample (*N* = 235) is formed of middle-aged (57.4%) and aging adults (41.6%), out of which 59.1% are females. The average age of the respondents is *M* = 51.13, *SD* = 5.954. Age has been measured as a continuous variable (“*Please state your age in full years*”). While middle-aged people are considered adults between 36 and 55 years old (Petry, 2002), aging adults are people over 50 years old (Mitzner et al., 2008; Renaud and Van Biljon, 2008). In the case of the present paper, middle-aged respondents are considered the ones between 40 and 50, and the aging ones are over 50. The age range of the present sample is 40–78 and these age limits are due to the sample selection process. The sample has been selected using the convenience sampling technique (Parker et al., 2019). Undergraduate communication students, on a voluntary basis and coordinated by the authors, sent the online questionnaires to their aging relatives. The questionnaires have been filled in between May and June 2021. Most of the respondents have an urban residence, have university studies, and have an income higher than 500 euros per month. Thus, there is the need to emphasize, from the very beginning, an over-representation of some demographic groups in the sample.

The table below (Table 1) summarizes the main demographic variables of the respondents.

**Table 1:** Demographics of the respondents.

Respondents’ characteristics	Frequency	Percentage (%)
	(*N* = 235)	
AGE		
40–50	135	57.40
51–78	100	42.60
GENDER		
Females	139	59.10
Males	96	40.90
RESIDENCE		
Urban	193	82.10
Rural	42	17.90
EDUCATION		
Primary school	1	0.40
Lower secondary education (8 classes)	5	2.10
Professional school	38	16.20
High school	0	0
Post-secondary school	53	22.60
University studies	138	58.70
INCOME		
Less than 300 euros	21	8.90
301–500 euros	54	23
501–700 euros	47	20
701–900 euros	53	22.6
More than 900 euros	60	25.5

### Measurements

The measurements have been adapted from the previous validated methodologies and developed based on the literature.

The previous experience with chatbots is measured by using the following variables. The respondents have been asked if they have heard about chatbots (HC) and if they have ever used them (UC; Lou and Remus, 2014) on scale from 1 to 7, where 1 = *never* and 7 = *very frequently*. Likewise, on a 7-point scale (1 = *nothing at all*, 7 = *a lot*), they have been inquired on their knowledge about chatbots (KC; Cui and Wu, 2019). The general attitude towards chatbots has been measured by using a simple question on liking this type of interaction (LKC; Edison and Geissler, 2003), on a 7-point scale, where 1 = *not at all* and 7 = *very much*.

The perceived ease of use (PEOU) scale, with seven items, is measured on 7-point scale, where 1 = *strongly disagree* and 7 = *strongly agree* (Van der Heijden et al., 2003; Venkatesh and Bala, 2008; Luo and Remus, 2014).

The perceive usefulness (PU) of interacting with chatbots uses nine items and it is measured on a 7-point scale, where 1 = *strongly disagree* and 7 = *strongly agree* (Venkatesh and Bala, 2008; Luo and Remus, 2014; O’Brien et al., 2018).

The enjoyment (ENJ) produced by interacting with a chatbot is measured through eight items, on a 7-point scale, where 1 = *strongly disagree* and 7 = *strongly agree* (Ryan, 1982; Ryan et al., 1983, 1990, 1991; Plant and Ryan, 1985; McAuley et al., 1987; Deci et al., 1994; Venkatesh and Bala, 2008).

The satisfaction (SA) with the interaction, or the output quality is measured through six items on a 7-point scale, where 1 = *strongly disagree* and 7 = *strongly agree* (Lou and Remus, 2014; Sherry et al., 2006; Venkatesh and Bala, 2008).

The effort (EFF) involved in chatbot interaction, or the objective usability, is measured using two items, on a 7-point scale, where 1 = *strongly disagree* and 7 = *strongly agree* (Ryan, 1982; Ryan et al., 1983, 1990, 1991; Plant and Ryan, 1985; McAuley et al., 1987; Deci et al., 1994; Venkatesh and Bala, 2008).

The perceived competence (COMP) of using a chatbot, or technology self-efficacy, is measured based on four items, on a 7-point scale, where 1 = *strongly disagree* and 7 = *strongly agree* (Ryan, 1982; Ryan et al., 1983, 1991; Plant and Ryan, 1985; McAuley et al., 1987; Ryan et al., 1990; Deci et al., 1994; Venkatesh and Bala, 2008).

The pressure (PRS) or anxiety generated by an interaction with a chatbot is measured by using 5 items on a 7-point scale, where 1 = *strongly disagree* and 7 = *strongly agree* (Ryan, 1982; Ryan et al., 1983, 1990, 1991; Plant and Ryan, 1985; McAuley et al., 1987; Deci et al., 1994; Venkatesh and Bala, 2008).

The perception of external control (PEC), or how much control one has on interacting with chatbots, is measured through two items on a 7-point scale, where 1 = *strongly disagree* and 7 = *strongly agree* (Venkatesh and Bala, 2008).

The subjective norms (SN) variable (the degree to which one perceives that people who are important to that person think he/she should use the system) is measured through two items on a 7-point scale, where 1 = *strongly disagree* and 7 = *strongly agree* (Venkatesh and Bala, 2008).

The behavioral intention (BI), or the intention to use chatbots in the future, is measured through four items on a 7-point scale, where 1 = *strongly disagree* and 7 = *strongly agree* (Lou and Remus, 2014).

The table below (Table 2) summarizes all the variable and items used and provides descriptive data for each item. The internal consistency has been computed using Cronbach’s α value. Overall, the results are satisfactory as all the constructs are higher than the acceptable threshold value of 0.6 (Fornell and Larcker, 1981; Nam et al., 2018). Factor loading has been assessed for each item. Some items have been removed due to low factor loadings (<0.05; e.g., PRS1, PRS3, and BI3).

**Table 2:** Measurements and items.

Variables	Items	*M* (SD)	Factor loading	Cronbach Alpha
Perceived ease of use (PEOU)	PEOU1: Learning to use this type of interaction is easy for me	4.65 (1.925)	0.847	0.964
	PEOU2: I find it easy to use this type of interaction	4.70 (1.945)	0.882
	PEOU3: I find this interaction to be flexible	4.52 (1.938)	0.74
	PEOU4: I find this type of interaction as being clear and understandable	4.66 (1.913)	0.893
	PEOU5: It is easy for me to become skillful at using this type of interaction	4.72 (1.934)	0.893
	PEOU6: This type of interaction does not require a lot of my mental effort	4.85 (1.939)	0.694
	PEOU7: I find this type of interaction as easy to use	4.76 (2.048)	0.886
Perceived usefulness (PU)	PU1: Using this type of interaction enables me to accomplish tasks more quickly	4.57 (1.995)	0.779	0.919
	PU2: Using this type of interaction improves my performance	4.18 (2.005)	0.869
	PU3: Using this type of interaction increases my productivity	4.02 (2.064)	0.89
	PU4: Using this type of interaction enhances my effectiveness	4.22 (2.057)	0.923
	PU5: Using this type of interaction makes it easier to do my work	4.49 (2.095)	0.866
	PU6: I find this type of interaction useful.	4.83 (1.981)	0.737
	PU7: I felt frustrated while using this interaction	5.17 (2.025)	0.755
	PU8: I found this interaction confusing to use	5.08 (1.910)	0.849
	PU9: Using this interaction was taxing	5.27 (1.909)	0.756
Enjoyment (ENJ)	ENJ1: I enjoy having this interaction very much	3.77 (1.954)	0.766	0.926
	ENJ2: This interaction is fun to do	3.28 (1.885)	0.806
	ENJ3: I think this is a boring interaction	4.66 (1.847)	0.806
	ENJ4: This interaction does not hold my attention at all	4.40 (1.994)	0.797
	ENJ5: I would describe this interaction as very interesting	3.63 (1.951)	0.875
	ENJ6: I think this interaction is quite enjoyable	3.60 (1.854)	0.919
	ENJ7: I think this interaction is quite captivating	3.40 (1.917)	0.887
	ENJ8: While seeing this interaction, I was thinking about how much I enjoyed it	3.10 (1.942)	0.854
Satisfaction/output quality (SA)	SA1: This interaction is a waste of time	5.09 (2.041)	0.575	0.898
	SA2: I would like to use this type of interaction more than I already do	3.71 (2.145)	0.665
	SA3: I am not satisfied with this type of interaction	4.92 (1.975)	0.585
	SA4: I enjoy using this type of interaction	3.62 (2.004)	0.798
	SA5: Using this type of interaction is personally satisfying	3.80 (1.969)	0.7
	SA6: I feel proud that I know how to use this interaction	3.85 (2.129)	0.67
Effort/objective usability (EFF)	EFF1: I put a lot of effort into this type of interaction	2.67 (1.853)	0.797	0.745
	EFF2: I try very hard on this type of interactions	2.93 (1.808)	0.797
Competence/self-efficacy on using chatbots (COMP)	COMP1: I think I am pretty good at this interaction	4.03 (1.965)	0.895	0.954
	COMP2: I feel competent in having such an interaction	4.01 (1.915)	0.906
	COMP3: I feel satisfied with my competence in such an interaction	3.90 (1.973)	0.842
	COMP4: This is an interaction that I could do very well on	4.27 (2.037)	0.879
Pressure (PRS)	PRS1: I do not feel nervous at all regarding this type of interaction	4.57(2.048)	0.246	0.795
	PRS2: I feel very tense regarding this type of interaction	5.20 (2.024)	0.783
	PRS3: I consider this interaction very relaxing	3.39 (1.933)	0.263
	PRS4: I am anxious regarding this interaction	5.52 (1.812)	0.78
	PRS5: I feel pressure regarding this interaction	5.38 (1.941)	0.811
Perception of external control (PEC)	PEC1: I have control over using a chatbot	3.76 (1.960)	0.852	0.827
	PEC2: I have the resources necessary to use a chatbot	4.14 (1.970)	0.852
Subjective norms (SN)	SN1: People who influence my behavior think that I should use chatbots	3.56 (2.040)	0.762	0.688
	SN2: I could conduct a complete activity with the chatbot if someone would show me how	4.48 (2.062)	0.762
Behavioral intention (BI)	BI1: I plan to use this interaction in the future	3.72 (2.012)	0.881	0.876
	BI2: I intend to continue using this type of interaction in the future	3.73 (2.032)	0.907
	BI3: I am not likely to use this type of interaction in the future	4.66 (2.085)	0.392
	BI4: I predict I will use this type of interaction in the future	4.21 (2.035)	0.8

## Results

From the point of view of previous experience with chatbot, more than 75% of the respondents have heard at least once about chatbots (*M* = 3.64, *SD* = 1.64) and around 70% have used this interaction at least on one occasion (*M* = 2.89, *SD* = 1.710). Table 3 presents this information in a comparative manner between women and men and between middle-aged and aging adults emphasizing on the upper part of the used scale. In this respect, age has been transformed into a dummy variable ([40–50] and [51–78] intervals). Overall, men seem to have more experience with chatbots. However, when it comes to age, the differences between middle-aged and aging adults are not that significant. Paradoxically, and probably due to social desirability, although a large majority of the respondents declare that they like chatbots (*M* = 4.12, *SD* = 2.006), only a small part of them have increased knowledge on them (*M* = 3.19, *SD* = 1.598).

**Table 3:** A comparative summary on the previous experience with chatbots.

Experience with chatbots	GENDER		AGE	
	Women	Men	Middle-aged adults	Aging adults
Heard about chatbots *(Frequently and very frequently)*	28.7% (*n* = 40)	45.8% (*n* = 44)	38.5% (*n* = 52)	32% (*n* = 32)
Used chatbots *(Frequently and very frequently)*	14.4% (*n* = 20)	25% (*n* = 24)	17.8% (*n* = 24)	20% (*n* = 20)
Have knowledge on chatbots *(Much and very much)*	12.9% (*n* = 18)	30.2% (*n* = 29)	19.2% (*n* = 26)	21% (*n* = 21)
Like chatbots *(Much and very much)*	43.8% (*n* = 61)	52.2% (*n* = 50)	47.4% (*n* = 64)	47% (*n* = 47)

**Figure 3:**
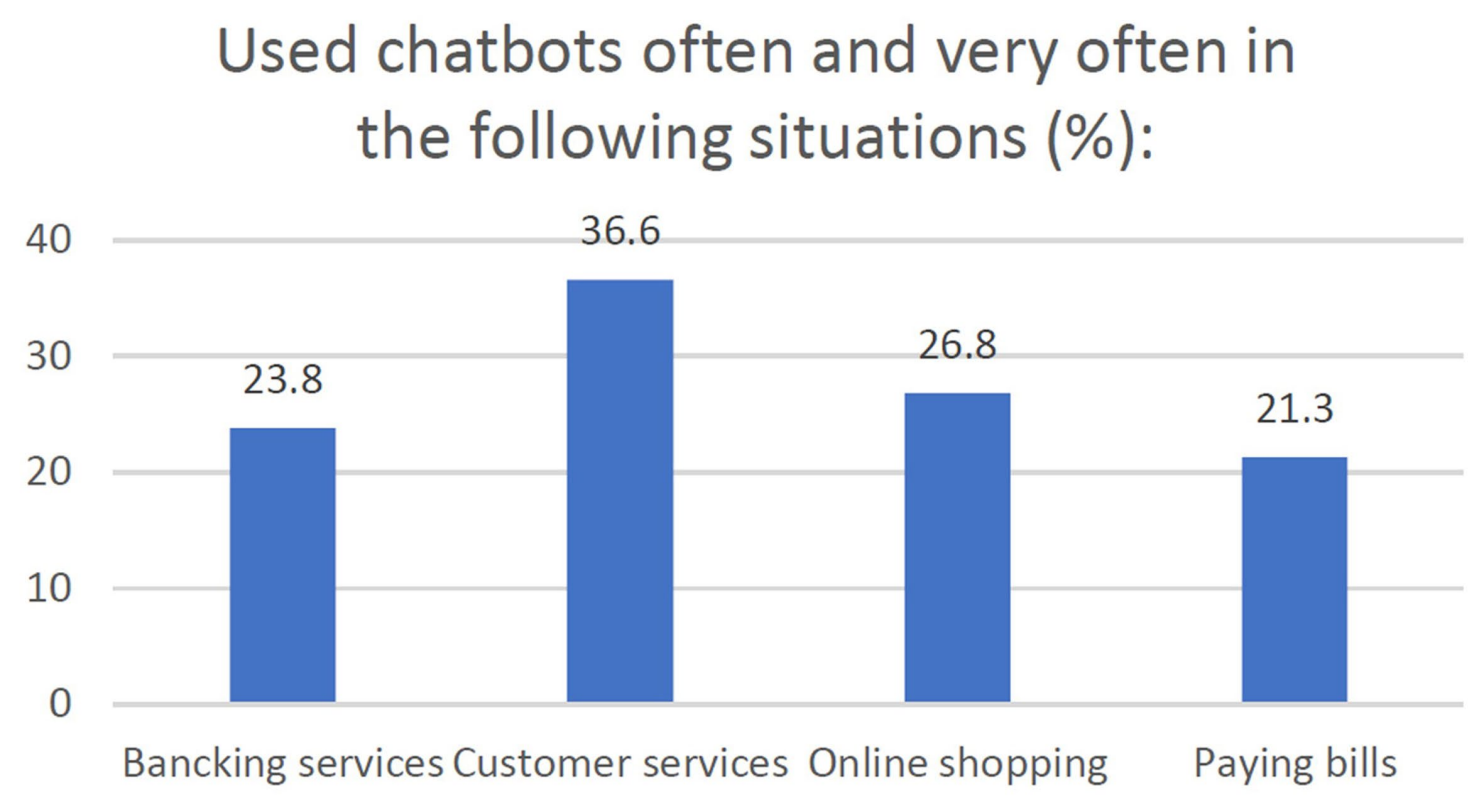
The use of chatbots for certain activities.

**Figure 4:**
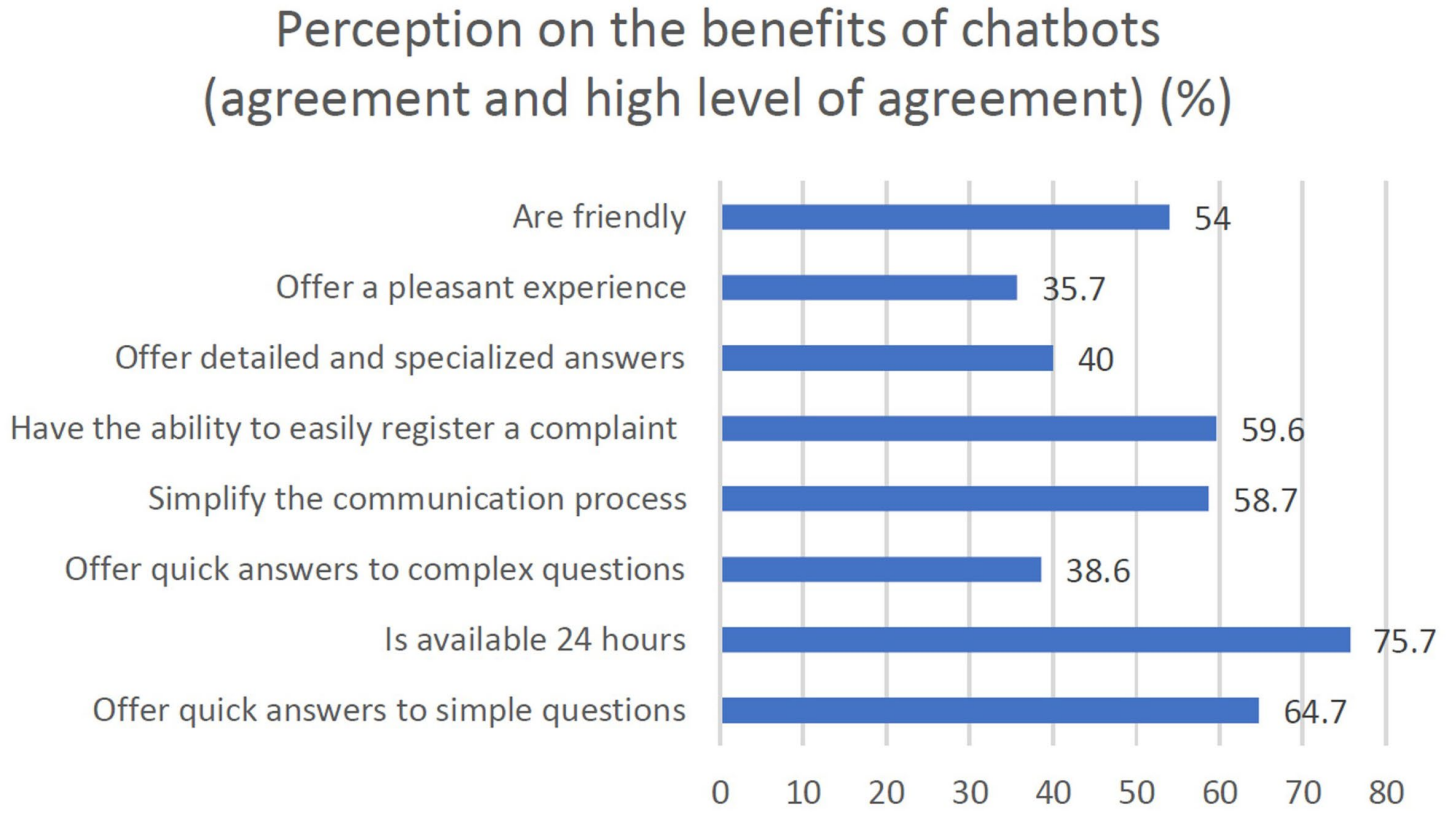
The perception of the benefits of chatbots.

As presented in Figure 3, the situations in which chatbots have been used regularly (Lou and Remus, 2014) are related to customer services and online shopping. These chatbots are similar in functionality and interaction and they are only tailored made for those domains. Furthermore, when it comes to benefits (State of Chatbot Report, 2018), as the Figure 4 shows, the respondents strongly appreciate chatbots mainly due to their availability (e.g., 24 h a day) and capabilities to solve problems (e.g., quick answers, register complains, simplify the communication process).

To better understand if there is an interaction between gender and age on the intentional behavior to use chatbots, a Two-way ANOVA analysis has been performed. The table below (Table 4) summarizes the descriptive statistics.

**Table 4:** The descriptive statistics for Two-way ANOVA [the dependent variable is Behavioral intention (BI)].

Gender	Age	Mean	*SD*	*N*
Feminine	40–50	3.9713	1.7017	87
	51–78	4.125	1.8816	52
	Total	4.0288	1.7659	139
Masculine	40–50	4.2865	1.6348	48
	51–78	4.0156	1.7979	48
	Total	4.151	1.7146	96
Total	40–50	4.0833	1.679	135
	51–78	4.0725	1.8334	100
	Total	4.0787	1.7425	235

The test of between-subjects effect shows no significant difference in mean behavioral intention between males and female [*F*(1, 231) = 0.191, *p* = 0.662] and between middle-aged and aging adults [*F*(1,231) = 0.62, *p* = 0.804].

Table 5 presents the correlation matrix between the main variables of the study. The most powerful relationships are going to be highlighted in the following rows. While perceived ease of use is strongly, positively, and significantly correlated with perceived usefulness, competence, and pressure, the perceived usefulness is strongly linked with enjoyment, pressure, and satisfaction. Finally, the more one likes chatbots, perceive them as being useful, enjoy them, and feel satisfaction while using them, the higher is the intention to use chatbots in the future. It is important to notice that gender is significantly correlated only with hearing on chatbots, using them, and have knowledge on them, men being more prone to that. However, the relationship is a weak one. Age is significantly and negatively correlated with perceived ease of use, perceived usefulness, effort, and pressure. Although the relationships are weak, further analyses should investigate more if middle-aged adults are perceiving chatbots as being easier to use and more useful, and if they indeed invest less effort and feel less pressure when using chatbots.

**Table 5:** Correlation matrix.

	HC	UC	KC	LKC	PEOU	PU	ENJ	COMP	EFF	PRS	SA	PEC	SN	BI	Gender
HC	1														
UC	0.577**	1													
KC	0.480**	0.497**	1												
LKC	0.096	0.280**	0.281**	1											
PEOU	0.313**	0.370*	0.310**	0.423**	1										
PU	0.216**	0.396**	0.228**	0.584**	0.782**	1									
ENJ	0.141*	0.338**	0.192**	0.686**	0.496**	0.725**	1								
COMP	0.291**	0.370**	0.383**	0.497**	0.765**	0.653**	0.598**	1							
EFF	−0.037	−0.039	−0.092	0.002	−0.216**	−0.164*	0.096	−0.077	1						
PRS	0.309**	0.371**	0.334**	0.476**	0.664**	0.608**	0.485**	0.657**	−0.372**	1					
SA	0.163*	0.330**	0.179**	0.615**	0.547**	0.781**	0.783**	0.605**	0.000	0.538*	1				
PEC	0.415**	0.439**	0.419**	0.265**	0.528**	0.432**	0.303**	538**	−0.101	0.454**	0.343**	1			
SN	0.058	0.129*	0.001	0.225**	0.184**	0.363**	0.400**	0.202**	0.189**	0.055	0.407**	0.314**	1		
BI	0.249**	0.395**	0.243**	0.609**	0.519**	0.771**	0.728**	0.584**	0.024	0.477**	0.823**	0.432**	0.418**	1	
Gender	0.146*	0.183**	0.206**	0.091	0.070	−0.005	−0.013	0.037	0.077	0.037	−0.029	0.117	0.041	0.036	1
Age	−0.161*	−0.073	−0.112	−0.084	−0.209**	−0.158*	−0.071	−0.116	0.185**	−0.208**	−0.120	−0.103	0.089	−0.056	0.115

**Correlation is significant at the 0.01 level; *Correlation is significant at the 0.05 level.

HC, heard of chatbots; UC, used chatbots; KC, knowledge on chatbots; LKC, like chatbots; PEOU, perceived ease of use; PU, perceived usefulness; ENJ, enjoyment; COMP, competence/self-efficacy; EFF, effort/objective usability; PRS, pressure; SA, satisfaction/output quality; PEC, perception of external control; SN, subjective norms; BI, behavioral intention.

A structural model assessment has been used to test the initial hypothesized relationships. The model-fit measurements have been used to evaluate the overall goodness of fit. In this respect, the following table (Table 6) summarizes the main indicators for the model and the standard values for a good fit. The standard values for a good fit are documented from Schumacker and Lomax (2004), Schreiber et al. (2006), and Shi et al. (2019). Overall, the data show that these indicators respect the recommended values for an acceptable fit. Thus, no modifications to the model have been done.

**Table 6:** The model fit summary.

**MODEL**	CMIN	Df	RMR	GFI	AGFI	NFI	RFI	IFI	TLI	CFI	RMSEA
Default model	21.57	14	0.063	0.989	0.891	0.99	0.912	0.996	0.967	0.996	0.048
	*p* = 0.088										
Recommended values for a good fit	*p* > 0.05	-	<0.08	>0.95	>0.90	>0.95	Close to 1	>0.90	>0.95	>0.90	<0.05

CMIN, chi-square value; Df, degrees of freedom; RMR, root mean square residual; GFI/AGFI, (adjusted) goodness OF fit; NFI, normed fit index; RFI, relative fit index; IFI, incremental fit index; TLI, Tucker Lewis Index; CFI, comparative fit index; RMSEA, root mean square error of approximation.

The study assesses the impact of different independent variables related to chatbots used on perceived ease of use, perceived usefulness, and behavioral intention. The following table (Table 7) summarizes the results.

**Table 7:** The summary of the hypotheses testing.

Hypothesis	Estimate	SE	CR	*P*	*R^2^*	Results
	Standardized (*β*)		(*t*)			
**H1a:** Enjoyment ➔ Perceive ease of use	0.007	0.075	0.11	0.912	0.644	Not supported
**H1b:** Satisfaction ➔ Perceive ease of use	0.121	0.07	1.83	0.067	Not supported
**H1c:** Effort ➔ Perceive ease of use	−0.138	0.047	−3.197	0.001	Supported
**H1d:** Competence ➔ Perceive ease of use	0.569	0.055	9.923	***	Supported
**H1e:** Pressure ➔ Perceive ease of use	0.079	0.049	1.6	0.11	Not supported
**H1f:** Perception of external control ➔ Perceive ease of use	0.124	0.045	2.71	0.007	Supported
**H2a:** Perceived ease of use ➔ Perceived usefulness	0.951	0.058	9.541	***	0.142	Supported
**H2b:** Subjective norms ➔ Perceived usefulness	0.806	0.15	4.434	***	Supported
**H3a:** Perceived usefulness ➔ Behavioral intention	0.908	0.113	7.397	***	0.571	Supported
**H3b:** Perceived ease of use ➔ Behavioral intention	−0.166	0.076	−1.579	0.114	Not supported
**H3c:** Subjective norms ➔ Behavioral intention	0.255	0.015	3.581	***	Supported
**H3d:** Knowledge on chatbots ➔ Behavioral intention	−0.014	0.053	−0.246	0.805	Not supported
**H3e:** Heard of chatbots ➔ Behavioral intention	0.103	0.068	1.883	0.06	Not supported
**H3f:** Use of chatbots ➔ Behavioral intention	−0.03	0.062	−0.491	0.623	Not supported
**H3g:** Like chatbots ➔ Behavioral intention	0.078	0.149	1.186	0.236	Not supported
**H4a:** Age ➔ Behavioral intention	0.047	0.172	0.997	0.319	Not supported
**H4b:** Gender ➔ Behavioral intention	0.015	0.067	0.345	0.73	Not supported

***p < 0.001.

The impact of enjoyment, satisfaction, and pressure on perceived ease of use of a chatbot are not significant (*p* > 0.05). Thus, hypotheses H1a, H1b, and H1e are not supported. However, the data show that effort (*β* = −0.138, *t* = −3.197, *p* = 0.001), competence (*β* = 0.569, *t* = 9.923, *p* < 0.001*)*, and perceived external control (*β* = 0.124, *t* = 2.710, *p* = 0.007) impact the perceived ease of use chatbots in a significant manner. Hence, H1c, H1d, and H1f are supported.

Perceived usefulness is positively and significantly impacted by both perceived ease of use (*β* = 0.951, *t* = 9.541, *p* < 0.001) and subjective norms (*β* = 0.806, *t* = 4.434, *p* < 0.001). Thus, H2a and H2b are supported by the data.

Behavioral intention to use a chatbot is not significantly impacted by perceived ease of use, age, gender, or the previous experience with the interaction (knowledge on chatbot, hearing of chatbot, use of chatbots, or like chatbots). Hence, hypotheses H3b, H3d, H3e, H3f, H3g, H4a, and H4b are not supported. However, perceived usefulness (*β* = 0.113, *t* = 7.397, *p* < 0.001) of a chatbot and subjective norms (*β* = 0.255, *t* = 3.581, *p* < 0.001) are positively and significantly impacting the behavioral intention of using this interaction in the future. Consequently, H3a and H3c are supported.

The square multiple correlation is *R^2^* = 0.644 for perceived ease of use. It means that 64% of the variance in the perceived ease of use is accounted by enjoyment, satisfaction, effort, competence, pressure, and perception of external control (however, only effort, competence, and perceived external control being significant). For the perceived usefulness, the square multiple correlation is *R^2^* = 0.142, which means that 14% of the variance in the perceived usefulness is explained by perceived ease of use and subjective norms. Finally, for the behavioral intention, the square multiple correlation is *R^2^* = 0.571. It means that 57% of the variance in the behavioral intention of using a chatbot is significantly accounted by perceived usefulness and subjective norms (perceived ease of use, age, gender, and experience variables not being significantly linked with behavioral intention).

**Figure 5:**
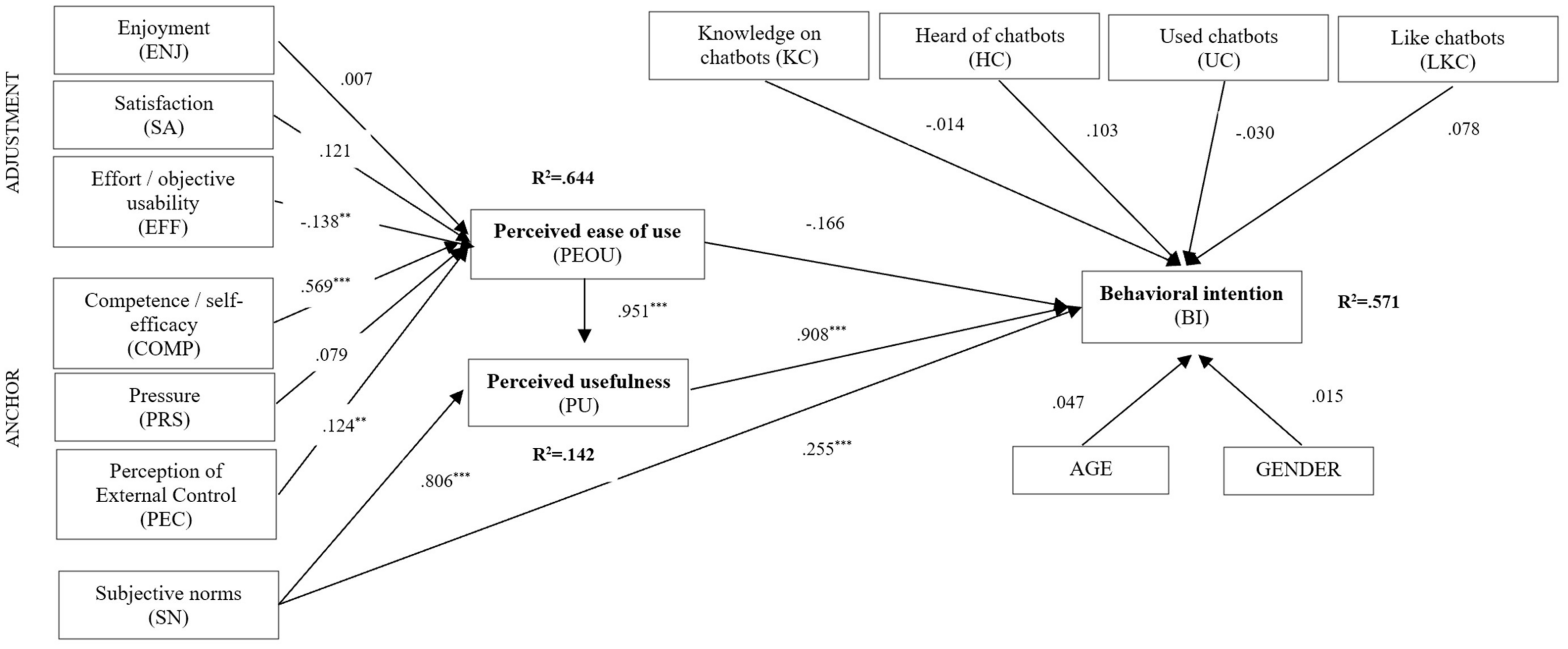
Results of the structural model.

The results of the structural model are summarized in the conceptual schema below (Figure 5).

## Discussion and conclusion

In a context in which the technological development is increasingly impacting the socio-economic environment and in which the aging population is already an acknowledged phenomenon, the present paper aims to better understand the way chatbots are perceived by middle-aged and aging adults in Romania. Since the existing literature on chatbots is mostly written in the computer science domain and/or with a strong focus on healthcare and assistive perspective, one of the original contributions of this paper resides in assessing the general view, not domain-specific, on chatbots later in life and from a social science standpoint. Moreover, since most devices and applications are designed by young specialists, the aging adults’ inputs are mandatory.

Starting from the COVID-19 pandemic situation, the need for digital solutions is emphasized (Valtolina and Marchionna, 2021). However, as older individuals are more reluctant to technology than youngsters (Edison and Geissler, 2003), investigating perception on technology later in life is paramount, not only thinking about the need for smart healthcare, but also considering daily routine activities, as paying a bill or shopping online.

By relying on complex theoretical models of technology acceptance, the present paper highlights the role of perceived ease of use of chatbots, their perceived usefulness, previous experience with chatbots and demographics on the behavioral intention to further use this type of interaction. A structural model has been used for hypotheses testing. The first assumption of the paper (H1) is introducing a wide range of variables as possible explanations for the perceived ease of use of chatbots. Chatbots are perceived as easy to use if the effort implied is low and if the users feel competent for this type of interaction. However, contrary to expectations, enjoyment, satisfaction, or pressure, although significantly correlated to perceived ease of use, are not directly influencing it. These results are contrasting a large set of findings on technology acceptance (Venkatesh, 2000; Zamora, 2017; Kim et al., 2018). Possible explanations might be related to the limits of the sample (in terms of number or over-representation of certain socio-demographical features, i.e., education), to the lack of knowledge on chatbots, or to a poor exposure to this type of technology. Thus, associating chatbots with different degrees of enjoyment, satisfaction, or pressure might be accomplished only after an adjustment time frame and an increased experience. Consequently, further investigation is needed on the way aging population perceive the ease of use of chatbots, a topic that is scarcely studied.

The second assumption (H2) of the paper implies that perceived usefulness of chatbots is predicted by the perceived ease of use of this technology and by the subjective norms. Data show that this hypothesis is supported. Thus, middle-aged and aging users consider that chatbots are useful mainly if they find them easy to be used, is people around them consider they should use this interaction, and if they are helped into this process. This conclusion is in line to the results of Venkatesh and Davis (2000) and Venkatesh and Bala (2008).

The third assumption (H3) is hypothesizing that behavioral intention is impacted by the perceived ease of use, perceived usefulness, subjective norms, and previous experience with chatbots. This assumption is based on the results of Venkatesh and Davis (2000), Lin et al. (2007), Renaud and Ramsay (2007), or Venkatesh and Bala (2008). The data show that, although there are significant correlations between all these variables, behavioral intention is only explained by the perceived usefulness of chatbots and by the subjective norms. In this respect, later in life, a more utilitarian perspective of technology and the role of peers seem to be more important.

Finally, the last hypothesis (H4) refers to the role of age and gender on the way further intentions to use chatbots are perceived. The data show that there are no relations between the way women or men, and middle-aged or aging adults are perceiving intentional behavior to use chatbots. This lack of difference seems to be acknowledged by Modahl (1999) that concludes that, mainly when it comes to internet use, the role of age and gender is reduced. In terms of age, the results can be explained by the low age average (*M* = 51.13) and by the fact that people above 60 years old are under-represented (8.2%) within the sample.

As studies on chatbots and aging adults are few and are mainly investigating reactions in the healthcare domain, this research is one of the first attempts to better understand the way chatbots in a not domain-specific context are perceived later in life. However, as some of the results are contradicting the existing theoretical models that explain technology acceptance, further inquiries are needed. One of the limits of the present paper is the small sample size and the convenience sampling method. Convenience samples are valuable for assessing attitudes and identifying new possible hypotheses that need further rigorous investigation (Galloway, 2005). In this respect, larger targets and a more in-depth approach should be investigated. Considering that age alone is not a socio-demographic sufficient variable to explain technology use (Loos, 2012), one important limit of the paper refers to the fact that aging adults that have rural residence, are less educated, and have low income are underrepresented in this study. To have a generalization potential of the results, future investigations should consider a better representation of the population within the sample for all the important socio-demographical variables. Likewise, as older people are not that comfortable with online questionnaires (Kelfve et al., 2022), doubled by a large range of statements investigated, the method might have created bias and desirability. It is very likely that an experimental setting would better fit the issue of chatbot testing. At the same time, a future comparative approach between the way not domain-specific chatbots and domain specific ones are perceived becomes of great interest. Another limit of the paper refers to single country study. Emphasizing the case of Romania and its specific digital literacy characteristics, the data cannot be generalized to any other socio-economic or cultural context. However, Romania, being ranked the last in digital skills among EU countries can serve as a valuable case-study for different techniques to overcome and improve the digital literacy gap. For a more comprehensive and global perspective, a comparative analysis with other countries is needed. Since assistive technologies are already largely used in developed countries, a best practice guide to reduce the economic and social gaps might be of great value.

The present paper’s contributions are twofold. On one hand, it is one of the first attempts to explore the middle-aged and aging adults’ perceptions on chatbots in a non-healthcare context in Romania. Considering that technology is increasingly present in our daily routine, this type of investigation is of great use. Furthermore, some of the variables included in other studies analyzing technology perception in different setups seem not that important in the case of chatbots’ use later in life and in the context of the least digitally educated country in EU. In this particular case, a lower degree of effort, an increased feeling of competence, external control and subjective norms, and a high utilitarian role of technology seem to be utmost factors in chatbots’ use. Finally, it is important to notice the inexistant differences at the age and gender levels. Thus, stereotypical perceptions should be overcome.

On the other hand, the implications of the present investigation echo at the managerial and business level. As training might be uncomfortable for aging individuals, developing chatbots that are intuitive and that do not need much preparation to be used might be a winning solution (Da Paixão Pinto et al., 2021). The practitioners that develop technological interactive systems should be aware of the needs of the aging adults. Thus, the take aways imply designing useful technologies that do not require effort in use and that provide feelings of competence for the user.

## Data availability statement

The raw data supporting the conclusions of this article will be made available by the authors, without undue reservation.

## Ethics statement

Ethical approval was not provided for this study on human participants because informed implicit consent was obtained when the survey has been conducted and the data was anonymized. The survey has been conducted online and the respondents opted in to participate. Written informed consent for participation was not required for this study in accordance with the national legislation and the institutional requirements.

## Author contributions

All authors listed have made a substantial, direct, and intellectual contribution to the work and approved it for publication.

## Conflict of interest

The authors declare that the research was conducted in the absence of any commercial or financial relationships that could be construed as a potential conflict of interest.

## Publisher’s note

All claims expressed in this article are solely those of the authors and do not necessarily represent those of their affiliated organizations, or those of the publisher, the editors and the reviewers. Any product that may be evaluated in this article, or claim that may be made by its manufacturer, is not guaranteed or endorsed by the publisher.
